# Preparation, Characterization, and Safety Evaluation of a Submicron Emulsion Processed Using High-Pressure Homogenization to Protect Bitter Melon Seed Oil

**DOI:** 10.3390/foods14050850

**Published:** 2025-03-01

**Authors:** Huiling Wang, Heng Guo, Xiaoyan Shuai, Yan Ma, Rui Zhang, Muci Wu, Jingren He, Jiayan Ling

**Affiliations:** 1 National R&D Center for Se-Rich Agricultural Products Processing, School of Modern Industry for Selenium Science and Engineering, Wuhan Polytechnic University, Wuhan 430023, China; 15629867033@163.com (H.W.); 13429973815@163.com (X.S.); yanma1227@sina.com (Y.M.); ruizhangwhpu@126.com (R.Z.); wumuci1991@163.com (M.W.); 2 Department of Pharmacy, Wuhan No. 1 Hospital (Traditional Chinese and Western Medicine Hospital of Wuhan), Tongji Medical College, Huazhong University of Science and Technology, Wuhan 430023, China; guoheng08@163.com; 3 Key Laboratory for Deep Processing of Major Grain and Oil, Ministry of Education, Wuhan Polytechnic University, Wuhan 430023, China; 4 Department of Traditional Chinese Medicine, Wuhan No. 1 Hospital (Traditional Chinese and Western Medicine Hospital of Wuhan), Tongji Medical College, Huazhong University of Science and Technology, Wuhan 430023, China

**Keywords:** bitter melon seed oil, submicron emulsion, formulation and preparation, physicochemical properties, safety evaluation

## Abstract

Bitter melon seed oil (BMSO), as a by-product of bitter gourd fruit processing, is rich in active ingredients and has unique medicinal potential. However, its solubility and dispersibility in water are poor when used directly. Therefore, this study aims to develop an eco-friendly submicron emulsion containing BMSO for intravenous injection and evaluate its safety. The BMSO submicron emulsion (BMSOSE) was prepared by high-pressure homogenization. The size, polydispersity index (PDI), ζ-potential, Turbiscan stability index (TSI), apparent viscosity, and morphology were characterized; in addition, an in vitro hemolysis test and acute toxicity test in mice were investigated in detail to evaluate the emulsion. The results demonstrated that the formulation and technological parameters of the BMSOSE were as follows: BMSO, 8% (*w*/*w*); egg yolk lecithin, 1.2% (*w*/*w*); F-68, 0.2% (*w*/*w*); pH, 5.0; homogenization pressure, 600 Pa; and number of homogenization cycle, 9. The obtained BMSOSE droplets exhibited a spherical shape with uniform size distribution with an average diameter of 221.3 nm, a PDI of 0.2, and a ζ-potential of −36 mV. There was no significant change in the fatty acid composition of BMSO and the BMSOSE. The safety tests demonstrated that the BMSOSE had no signs of hemolysis and had no toxicity to mice with LD50 > 64 mL/kg. This study provides a foundation for further development of BMSO and its preparations.

## 1. Introduction

Bitter melon, a member of the Cucurbitaceae family and commonly known as bitter gourd or karela, thrives in humid and subtropical regions worldwide [1]. It is consumed as a vegetable in China and other Asian countries [2]. Various parts of this plant have been recognized for their medicinal properties [3,4]. Bitter melon seeds are rich in proteins, fatty acids, agglutinin, flavonoids, lycopene, and other active ingredients, which have significant antioxidative, antiviral, antibacterial, and antitumor effects [5,6,7]. With the advances in research on bitter melon, more studies have revealed that bitter melon seed oil (BMSO) contains a large amount of unsaturated fatty acids. It is worth noting that BMSO contains relevant amounts of the conjugated fatty acid α-eleostearic acid (C18:3 9*c*11*t*13*t*), a positional and geometric isomer of α-linolenic acid, which has been demonstrated to have multiple beneficial effects, such as antioxidant, anticancer, anti-inflammatory, anti-obesity, anti-diabetic, and anti-atherosclerotic effects [2,8,9,10,11]. Therefore, the development and utilization of BMSO have attracted increasing attention. However, α-eleostearic acid in BMSO is easily oxidized and isomerized under the catalysis of light and trace metals. The inherent instability coupled with its strong unpleasant flavor and taste significantly hinder the widespread application of BMSO. Therefore, it is imperative to explore novel strategies for stabilizing BMSO.

A submicron emulsion (SE) is formed by dispersing emulsion drops ranging from 0.1 to 1 μm in size into another liquid. Since the size of SE drops is between emulsion and microemulsion, the oil is wrapped in tiny emulsion particles through emulsification, which enables them to avoid the influence of the surrounding environment on the drug. Moreover, the emulsion has a certain passive targeting effect due to its small droplet size and can improve the stability of fatty acids, protecting them from oxidation, reducing the probability of drug decomposition and deterioration, blood vessel irritation, and adverse reactions of solventizing [12,13]. Ma et al. [14] found that the SE delivery system improved the hydrophilicity and lipophilicity of ursodeoxycholic acid (UA), increased the speed and degree of drug dissolution in the intestine, and significantly enhanced the bioavailability of UA in rats treated with an oral UA submicron emulsion compared to those treated with crude UA.

Furthermore, in recent studies, intravenous emulsions are widely used for lipid soluble substances, such as paclitaxel and vitamins. Through intravenous administration, the active substance can quickly enter the blood circulation, achieving sustained and controlled release of bioactive substances; reduce the adverse effects of traditional dosage forms; decrease vascular stimulation and inflammatory response; and exhibit certain passive targeting [15,16,17,18]. Moreover, as an efficient nutritional supplement, injectable emulsions are commonly used to enhance specific nutrients in food, such as vitamins and minerals, and for special medical purposes, such as providing nutritional support for postoperative recovery and malnourished patients, such as fat emulsions. And in the field of sports, BCAA injection emulsion is used by athletes for rapid muscle recovery. It is also used in intestinal health, such as for the delivery of probiotics. However, the options for emulsifiers in intravenous injection are limited, with pluronic F68, soya lecithin, and egg yolk lecithin being common choices.

At present, many studies focus on the extraction methods and physiological activity of bitter melon seed oil, but there is a lack of research on the application of BMSO. Given the biological activity of BMSO and the advantages of submicron emulsion, we investigated the preparation, characterization, and safety evaluation of a BMSOSE to provide the experimental reference and basis for developing BMSO-based products.

## 2. Materials and Methods

### 2.1. Chemicals and Reagents

BMSO was obtained from Deyang Sun Create Technology Co., Ltd., Deyang, China; it is produced by a supercritical CO_2_ extraction process. Glycerin was purchased from Hunan Erkang Pharmaceutical Co., Ltd., Changsha, China. Egg yolk lecithin was purchased from Nanjing Will Pharmaceutical Industry, Nanjing, China, while sodium oleate was purchased from Aweitao Pharmaceutical Technology Co., Ltd., Shanghai, China; Pluronic F68 was procured from Jiangsu Bomeida Life Science Co., Ltd., Suzhou, China; water for injection was purchased from Thermo Fisher Scientific (China) Co., Ltd., Shanghai, China; Hydrochloric acid (AR), eosin Y (water-soluble) (AR), xylene (AR), neutral gum, and embedded paraffin wax were obtained from the Sinopharm group. Sterile defibrillated sheep blood (BR) was purchased from Nanjing Ason Biotechnology, Nanjing, China. Paraformaldehyde (4%; AR) was procured from Biosharp Biological Co., Ltd., Shanghai, China. Normal (0.9%) saline was purchased from Wuhan Binhu Double Crane Pharmaceutical Co., Ltd., Wuhan, China. Hematoxylin was obtained from Sigma-Aldrich (Shanghai) Trading Co., Ltd, Shanghai. Creatinine (CRE) determination kit (BR), glutamic-oxalacetic transaminase (AST/GOT) test box (BR), and alanine aminotransferase (ALT/GPT) test box (BR) were purchased from Nanjing Jiancheng Institute of Biological Engineering, Nanjing, China. Sterile defibrinated sheep blood was purchased from Beijing Solarbio science & technology Co., Ltd., Beijing, China.

### 2.2. Animals

ICR mice were SPF grade, 4 weeks old, and weighed 20.0 g ± 2.0 g; a total of 50 mice were purchased from the Experimental Animal Center of Huazhong University of Science and Technology (Wuhan) (the license number of laboratory animal production: SCXK (Jing) 2019-0010).

ICR mice were housed in a specific pathogen-free laboratory, Experimental Animal Center of Huazhong University of Science and Technology [Laboratory Animal License number: SYXK (E) 2021-0057], at a temperature of 21–25 °C and a relative humidity of 45.0–55.0%. The mice were allowed to adapt to the environment for at least one week before the experiments. The performance standards of the experiments were in accordance with the Regulations on the Management of Experimental Animals (Order No. 2 of the National Science and Technology Commission of China, revised 2017). Furthermore, the European Community guidelines (EEC Directive of 1986: 86/609/EEC) were implemented on all the procedures and animal care. All experimental procedures involving animals were approved by the Ethics Committee of Huazhong University of Science and Technology (Wuhan, China).

### 2.3. Preparation of the BMSOSE

A specific amount of BMSO, egg yolk lecithin, pluronic F68, and glycerin was dispersed in an appropriate amount of water for injection to form the aqueous phase, and BMSO was the oil phase. The two phases were preheated to 70 °C, respectively; the oil phase was slowly added to the water phase and placed in a high-speed shear mixer (Ultra-Turrax, model T18, IKA, Werke, Germany) at 10,000 r/min for 5 min. The pH was adjusted, and it was then added to a high-pressure homogenizer (model AH-2010, ATS Industrial Systems Co., Ltd., Shanghai, China), homogenized several times under a certain pressure, and then filtered and sterilized with a 0.22 μm microporous membrane. Finally, the obtained lotion (i.e., BMSOSE) was injected into an ampoule of 25 mL, and then the ampoule bottle was fused and sealed.

Through a single-factor experiment, the addition amounts of BMSO (4–12%), egg yolk lecithin (0.6–1.5%), and pluronic F68 (0–0.4%); the pH of the emulsion (4–9); and the high-pressure homogenization times (3–13) in the preparation process of the BMSOSE were optimized.

### 2.4. Characterization of BMSOSE

The size, polydispersity index (PDI), and ζ-potential were measured by dynamic light scattering (DLS) using a Zetasizer Nano-ZS (Zetasizer Nano ZS 90, Malvern Instruments Ltd., Malvern, UK).

Immediately after the BMSOSE formation, measurements were taken at 25 °C with an equilibrium time of 60 s. Before size analysis, the emulsion was diluted with ultrapure water at a dilution factor of 1:100 (sample-to-water) to avoid multiple scattering effects. The measurements were carried out in triplicate. Values of 1.45 and 0.001 were used for the refractive and absorption indexes of BMSO, respectively. The refractive index and dielectric constant of the water dispersant were set at 1.33 and 78.5, respectively.

The Turbiscan stability index (TSI) was measured using the Turbiscan Lab Expert (Formulation, Toulouse, France). Twenty milliliters of emulsion were placed in special Turbiscan sample bottles that were scanned at 25 °C every 1 min for 10 min, and then once every 5 min for 50 min, during which the TSI changes in the emulsion were observed.

### 2.5. Apparent Viscosity

The apparent viscosity of the BMSOSE was determined using the TA rheometer (Discovery DHR-2 rheometer, TA instruments, New Castle, DE, USA). The BMSOSE (~1.5 mL) was placed on the plate, and the flow temperature slope mode was selected. Flow scanning mode was selected, and the measurement parameters were as follows: the shear rate ranged from 1 to 100 s^−1^, and the temperature was set at 25 °C [19].

### 2.6. Morphology Observation

The morphology of the BMSOSE was observed using a transmission electron microscope (TEM) (model JEM-2100, JEOL, Akishima, Japan). The SE of BMSO was treated using the negative dyeing method. After diluting 100 times, 1% phosphotungstic acid was used to negate the emulsion. After dyeing, a drop of BMSO was placed on the copper net in the TEM and dried at 25 °C, and images were captured about 15–20 min later under the TEM.

### 2.7. Fatty Acid Composition Determination

De-emulsion: Take 10 mL of the BMSOSE, add 1.0 g of anhydrous sodium sulfate, shake appropriately and mix well, place it in a beaker with 70 °C water for ultrasonic for 20 min, and then centrifuge at 6000 r/min for 20 min. The water phase and oil phase should be able to separate.

Fatty acid methylation: About 0.3 g of BMSO was added to 8 mL of 2% potassium hydroxide–methanol solution. The resulting solution was refluxed in a water bath at 80 °C ± 1 °C until the oil droplets disappeared. Seven milliliters of methanol solution containing 15% boron trifluoride was added to the refluxing solution, and the reflux was continued for 2 min in a water bath at 80 °C ± 1 °C. Next, the reaction was terminated, and the refluxing solution was cooled to room temperature.

To a flask, 6 mL of n-hexane was added, and then the solution was shaken for 2 min. Two milliliters of saturated sodium chloride aqueous solution was added to the above solution and allowed to stand for stratification. The upper n-hexane extract was separated, and 3 g of anhydrous sodium sulfate was added. After 1 min of shaking, the solution was left standing for 5 min. The upper solution was separated into the injection flask for determination.

Methyl esterification of the α-eleostearic acid standard was prepared using the same procedure as above.

The experimental conditions of the GC–MS system were as follows: Gas chromatographic column was HP-88, 100 m × 0.25 mm × 0.20 μm; carrier gas was 99.999% high-purity nitrogen in a constant current mode; the column flow rate was 1 mL/min; the injection volume was 1 μL without diversion; and the temperature of the injector was 250 °C. The temperature was set at 150 °C at 0 min, then programmed to 190 °C for 5 min at 4 °C/min, and raised to 230 °C at a rate of 2 °C/min for 10 min. The mass spectrum conditions were as follows: Ionization mode EI+, emission current of 200 VA; electron energy of 70 eV; interface temperature of 250 °C; ion source temperature of 200 °C; and the detection voltage was 350 V. The mass spectrometry library was NIST11.L.

### 2.8. Safety Verification of the BMSOSE

#### 2.8.1. Hemolysis Test In Vitro

Preparation of 2% erythrocyte suspension [20]: The sterile defibrinated sheep blood was taken, and 50 mL of normal saline (0.9% sodium chloride solution) was added, mixed well, and then centrifuged at 2000 rpm for 15 min. The supernatant was removed, and the precipitated red blood cells were washed thrice with normal saline until the supernatant became clear and had no red color. The obtained red blood cell solution was combined (2 mL) and diluted with normal saline (100 mL) to prepare a 2% suspension.

Hemolysis test: Five clean glass (10 mL) test tubes were numbered 1, 2, 3, 4, and 5, and the procedure was carried out according to the experimental design detailed in Table 1. Tube No. 1 and 2 contained the test solutions, and No. 3, 4, and 5 were the negative, positive, and blank control solutions, respectively. After the preparation of the individual solutions, each tube was gently mixed and incubated in a water bath at 37 °C. After 6 h, hemolysis in each tube was observed and recorded according to the judgment criteria shown in Table 2.

#### 2.8.2. Acute Toxicity Test

A total of 50 SPF ICR mice, aged 4 weeks and weighing 20 ± 2 g, were used for the acute toxicity tests. After adaptive feeding for one week, the mice were randomly divided into five groups, normal, low dose, low to medium dose, medium to high dose, and high dose, with 10 mice in each group (5 males and 5 females). Before the experiment, the mice underwent fasting for 12 h. The normal group was intraperitoneal injected (i.p.) with saline solution; the injection dose was 64 mL/kg BW. The low dose, low to medium dose, medium to high dose, and high dose groups of mice were i.p. with the prepared BMSOSE, with injection doses of 8, 16, 32, and 64 mL/kg, respectively. All the doses were divided equally into three injections. The interval between two injections was 6 h, and the stress response of the mice was observed regularly in the first 24 h after injection to check for signs of tremor, convulsion, diarrhea, drowsiness, coma, and other symptoms, and timely measures were taken, and their reasons were analyzed. The body weight of the mice was recorded for 15 days.

On the 15th day of the experiment, all the tested animals were weighed, and their weights were recorded. Next, their eyeballs were harvested, and blood was collected from the mice. Mice were dissected after humane euthanasia. After the blood was placed at 4 °C for 4 h, the supernatant was extracted by centrifugation at 3000 rpm for 10 min. The expression of GPT, GOT, and CR was detected using a microplate analyzer (Meiguo Molecular Instruments Co., Ltd., Shanghai, China). After dissection, the heart, liver, spleen, lung, and kidney were collected, and the anatomical findings were recorded in detail. After removing excess adipose tissue, all other tissues and organs were fixed with 4% paraformaldehyde, sectioned, and stained. The pathological changes in the sections of the organs of the mice were observed under a light microscope (BX53 biological microscope, Olympus Corporation, Shinjuku, Japan) [21].

### 2.9. Statistical Analysis

All results were subjected to an analysis of variance using the SPSS statistical package (ver. 23, SPSS Inc., Chicago, IL, USA). Significant differences between mean values (*p* < 0.05) were determined using LSD and Tukey’s test. The results were reported as the means ± standard deviation.

## 3. Results and Discussion

### 3.1. Influence of BMSO Concentration on the BMSOSE

**Figure 1:**
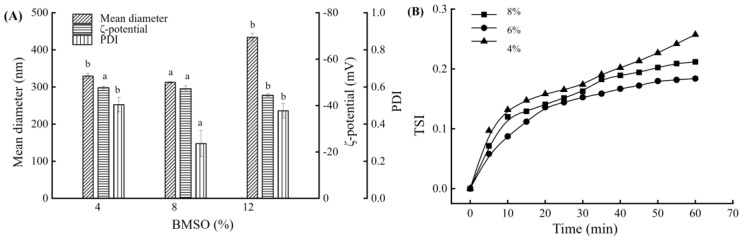
Mean diameter, PDI, and ζ-potential (**A**) of the BMSOSE with different BMSO concentrations. Different lower-case letters indicate significant differences between the BMSO concentration values (*p* < 0.05). TSI of the BMSOSE containing different BMSO concentrations (4–12% (*w*/*v*)) (**B**).

The mean diameter, PDI, ζ-potential, and TSI measurements of the SE containing different BMSO concentrations (4–12% (*w*/*v*)) are shown in Figure 1.

It was observed that, with an increase in BMSO concentration, the mean diameter and PDI of the emulsion decreased initially and then increased, but the ζ-potential continued to decrease. When the concentration of BMSO was 8%, the emulsion exhibited a more uniform particle size distribution and a more stable TSI than when the BMSO concentration was 4%. This phenomenon was probably due to the higher oil loading, resulting in more and smaller homogenized droplets. This, in turn, provided the emulsifier with a larger adsorbable surface, making the interfacial film surrounding the oil droplets more stable and the coalescence force stronger [22]. However, when the BMSO concentration increased to 12%, larger particle sizes were formed, indicating that the emulsifier could not completely embed the excessive oil phase. This resulted in particle agglomerations as well as adversely affected the particle size and stability of the emulsion. Therefore, an SE containing 8% (*w*/*v*) BMSO was selected for subsequent analyses.

### 3.2. Influence of Emulsifier Concentration on the BMSOSE

**Figure 2:**
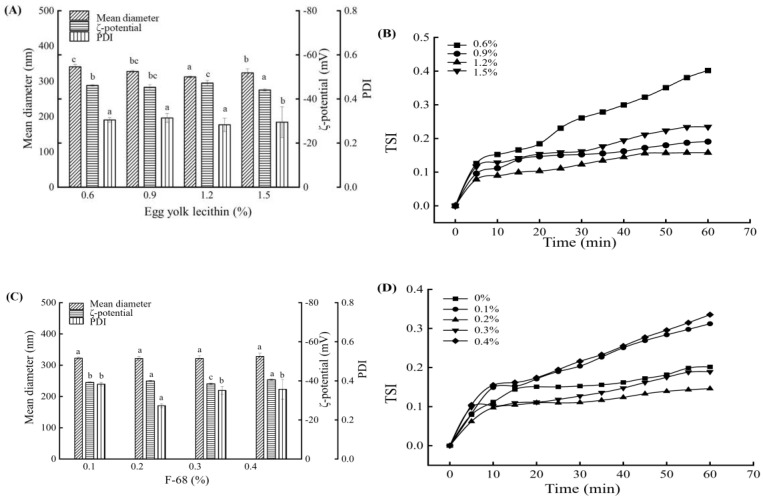
Mean diameter, ζ-potential, and PDI of the BMSOSE containing different concentrations of egg yolk lecithin (0.6–1.5% (*w*/*v*)) (**A**), and F-68 (0–0.4% (*w*/*v*)) (**C**). TSI of the BMSOSE containing different concentrations of egg yolk lecithin (0.6–1.5% (*w*/*v*)) (**B**) and F-68 (0–0.4% (*w*/*v*)) (**D**). Different lower-case letters indicate significant differences between the BMSO concentration values (*p* < 0.05).

The mean diameter, PDI, ζ-potential, and TSI measurements of the SE containing different concentrations of egg yolk lecithin (0.6–1.5% (*w*/*v*)) and F-68 (0–0.4% (*w*/*v*)) are shown in Figure 2.

Lecithin promotes overall solubility by burying the hydrophobic amino acids or exposing more charges, thereby increasing the emulsifying ability [23]. Egg yolk lecithin has a similar structure to animal cell membranes and has been widely used in emulsion preparation. An emulsion with a droplet size of 308 nm was obtained at an egg yolk lecithin concentration of 1.2% (*w*/*v*); the ζ-potential was −46.17 mV, and the PDI was 0.283. These results indicated that the emulsion containing a low concentration of egg yolk lecithin could not fully emulsify BMSO and showed an uneven distribution on the oil–water interface of the emulsion to form a solid and compact emulsifier interface film. When the content of lecithin was >1.2% (*w*/*v*), the relative balance among the droplets was destroyed with the increase in emulsifier content, and the egg yolk lecithin not adsorbed to the water–oil interface was dispersed into the continuous phase, causing droplet flocculation. Therefore, a concentration of 1.2% (*w*/*v*) egg yolk lecithin was selected for subsequent analysis.

An emulsifier interface film formed with lecithin is not sufficiently stable. It is difficult to form a stable emulsion with a single emulsifier, but it can be combined with phospholipids. The commonly used emulsifiers include tween and poloxamer; however, tween is potentially unsafe, hemolytic, and allergenic [24]. As shown in Figure 2, upon the addition of F-68, a nonsignificant trend in change was observed in the average droplet size of the emulsion, and the ζ-potential decreased slightly. The PDI reached the minimum when the F-68 mass fraction was 0.2%, and the emulsion stability was slightly improved. Except for 0.1%, the grain size distribution of the emulsion was unimodal, and the distribution was narrow. The growth trend of all TSI began to slow down after 10 min and was gradually stable with increasing time. Therefore, after a comprehensive consideration, the mass fraction of F-68 was selected as 0.2%, the average particle size of the emulsion was 321.50 nm, the ζ-potential was −39.93 mV, and the PDI was 0.273. Therefore, the optimal emulsifier concentration was as follows: the yolk lecithin content was 1.2%, and the F-68 content was 0.2%.

### 3.3. Influence of pH on the BMSOSE

**Figure 3:**
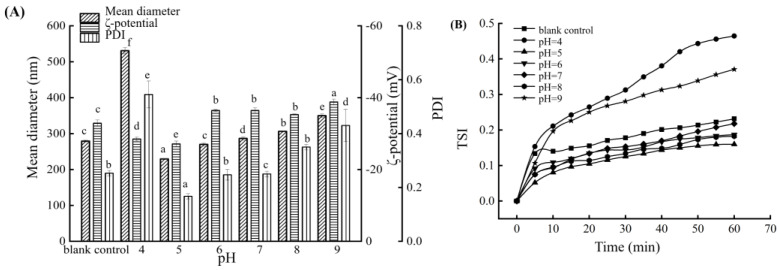
Mean diameter, ζ-potential, and PDI of the BMSOSE with different pH (4–9) (**A**). TSI of the BMSOSE with different pH (4–9) (**B**). Different lower-case letters indicate significant differences between the BMSO concentration values (*p* < 0.05).

Since high-pressure homogenization and the sterilization process can affect the pH stability of the emulsion and human tolerance to intravenous injections ranges from pH 4 to 11 [25], this study explored the influence of pH in the range of 4–9 on the submicron emulsion of BMSO. According to the optimal preparation conditions, the initial pH value of the emulsion was 4.34. As shown in Figure 3, when the pH was adjusted to 4, the average droplet size and PDI of the emulsion increased significantly (*p* < 0.05), the droplet size distribution was uneven, and TSI continued to increase. When the pH was between 5 and 9, the average droplet size, ζ-potential, and PDI showed a trend of an initial decrease and subsequent increase. The droplet size distribution was the most uniform, and the TSI growth trend was the slowest. The pH reflects the concentration of H^+^ ions in the system; as the H^+^ ions gradually increase, excess H^+^ ions penetrate the adsorption layer of the emulsion particles to neutralize the negative charge carried on the surface of the particles. Therefore, the amount of charge carried by the particles gradually decreases, and the emulsion flocculates with the increase in particle size. Therefore, we chose to adjust the pH to 5. The average droplet size of the emulsion was 229.40 nm, and the ζ-potential was −27.13 mV, while the PDI was 0.167.

### 3.4. Influence of Homogenization Time on the BMSOSE

**Figure 4:**
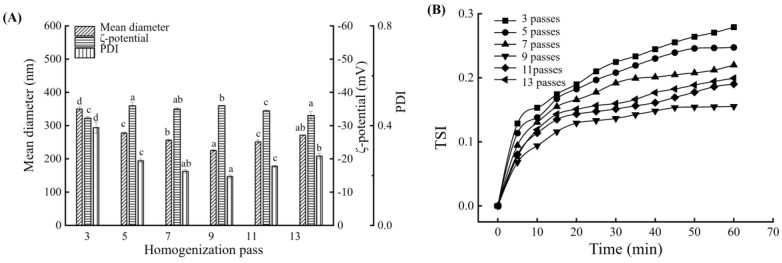
Mean diameter, ζ-potential, and PDI of the BMSOSE prepared with different homogenization passes (3–12) (**A**). TSI of the BMSOSE prepared with different homogenization passes (3–12) (**B**). Different lower-case letters indicate significant differences between the number of passes (*p* < 0.05).

Figure 4 shows the mean diameter, ζ-potential, PDI, and TSI of the BMSOSE under different homogenization passes. High-pressure homogenization can improve the physical and microbiological stability of the emulsion system [26]. Preliminary results showed that homogenization at 600 bar produced emulsions with fine droplet sizes and low PDI values. Furthermore, the TSI and ζ-potential also showed relatively stable emulsions at nine homogenization passes. Appropriate pressure and several passes of homogenization can produce smaller particle sizes and more stable emulsions. When the homogenization pressure and the number of homogenizations are high, the kinetic energy of the droplets increases, and the collision accelerates, leading to the merger of particles to a certain extent; that is, the particle size of the emulsion does not decrease. Excessive energy input makes the emulsion system overload, which reduces the stability of the emulsion [14].

Therefore, a homogenization pressure of 600 bar and nine homogenization passes were the chosen parameters in this study. Under these conditions, the mean size of the emulsion was 225 nm, the ζ-potential was −36 mV, and the PDI was 0.196.

### 3.5. Physical Properties of the BMSOSE

**Figure 5:**
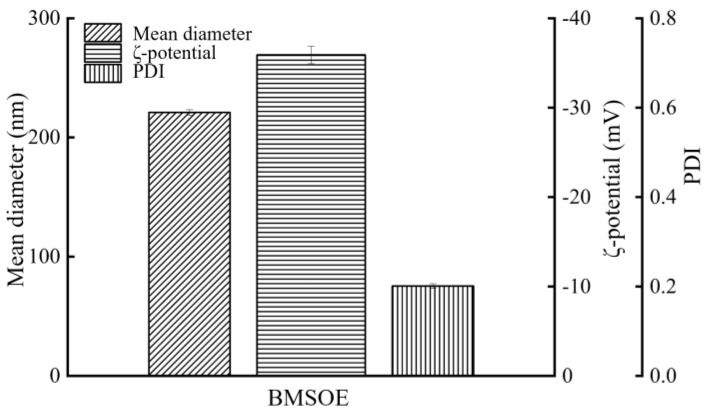
Mean diameter, ζ-potential, and PDI of the BMSOSE.

**Figure 6:**
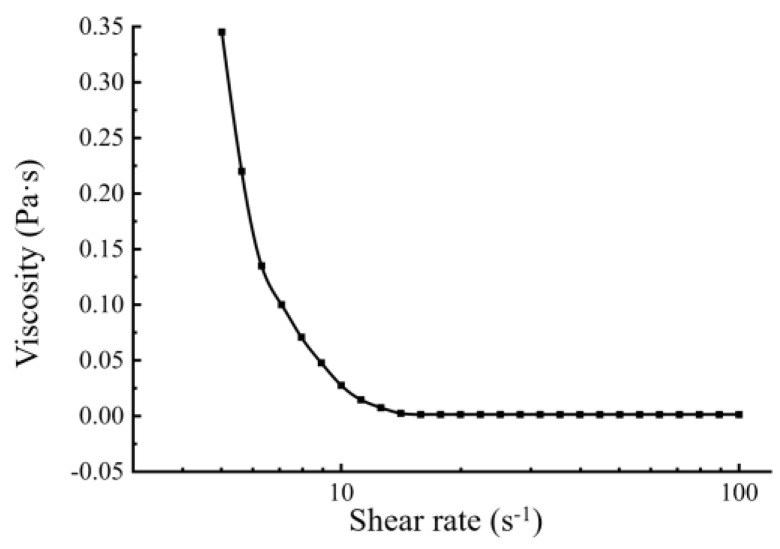
Steady-shear viscosity of the BMSOSE.

**Figure 7:**
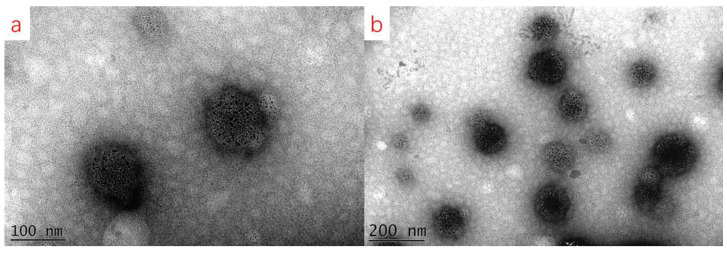
TEM image of the BMSOSE. Scale bar:100nm (**a**); scale bar:200nm (**b**).

Under optimal conditions (i.e., 8% (*w*/*v*) BMSO, 1.2% (*w*/*v*) egg yolk lecithin, 0.2% (*w*/*v*) F-68 content, pH 5.0, homogenization pressure of 600 bar, and nine homogenization passes), the obtained BMSOSE was characterized as mean diameter, PDI, ζ-potential, viscosity, and microstructural diagram (Figure 5, Figure 6 and Figure 7). The mean diameter of the emulsion droplet was about 221 nm, with a stable PDI around 0.201 and a ζ-potential of −35.9 mV. The emulsion particle size distribution was uniform, while the appearance was a milky-white emulsion liquid with good fluidity, with no oil and wall hanging phenomenon.

The rheological properties are used as a quality control tool for fat emulsion. The viscosity change curve of the emulsion system is used to characterize the polymerization degree of emulsion to some extent and further reflect its dynamic stability. The rheological properties of a fat emulsion depend on many factors, such as the type of emulsifier and oil phase, the proportion of continuous phase to dispersed phase, and particle size and distribution [27]. Figure 6 shows that the viscosity of the emulsion decreased sharply with increasing shear rate, indicating that the emulsion was a non-Newtonian fluid. When the emulsion has a larger viscosity, it is beneficial to slow down the floating speed of the droplet and maintain better stability.

As illustrated in Figure 7, the droplets of the BMSOSE were spherical, and the average particle size of the emulsion was small. Although there were still a few small droplets, most SE droplets were uniform in size and shape.

### 3.6. Fatty Acid Composition of the BMSOSE

As seen in Table 3, the fatty acid composition of BMSO changed after emulsification. For example, the contents of oleic acid, linoleic acid, and α-eleostearic acid decreased slightly. This may be due to the oxidation loss of these substances in the emulsion caused by homogenization under high pressure. However, in general, a more stable emulsion system can be formed as an SE after the high-pressure homogenization of BMSO, in which the functional components can be quickly and stably emulsified in the nanosystem, and the loss of BMSO in the process of production, processing, transportation, and storage can be reduced.

### 3.7. Hemolysis Testing of the BMSOSE

**Figure 8:**
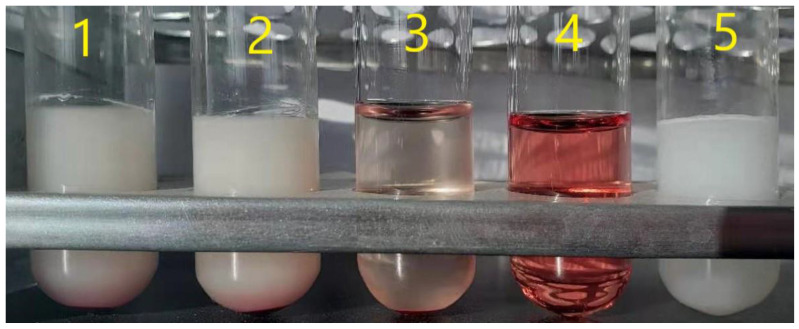
Hemolysis test results of the BMSOSE. No. 1–2 are the test solutions, No. 3 is the negative control solution, No. 4 is the positive control solution, and No. 5 is the blank control solution.

The composition of an SE preparation is complex. The unsaturated bond of the lecithin molecule is easily oxidized to form the structure of the diene bond, leading to hemolysis toxicity [28]. Therefore, it is necessary to test the hemolysis of the BMSOSE. As seen in Figure 8, all the red blood cells were sinking, and stratification was clear in samples No. 1 and 2. The bottom red blood cells were dispersed again after gently shaking three times, indicating no red blood cell agglutination.

There was no apparent red color in the upper layer, and no significant difference was observed compared with the liquid phase of sample No. 3 (negative control). Sample No. 4, the positive control group, exhibited noticeable RBC rupture and a clear red solution, which was significantly different from the solution of samples No. 1–3. Therefore, it can be concluded that there is no hemolysis in the BMSOSE, ensuring its safety.

### 3.8. Acute Toxicity of the BMSOSE

**Figure 9:**
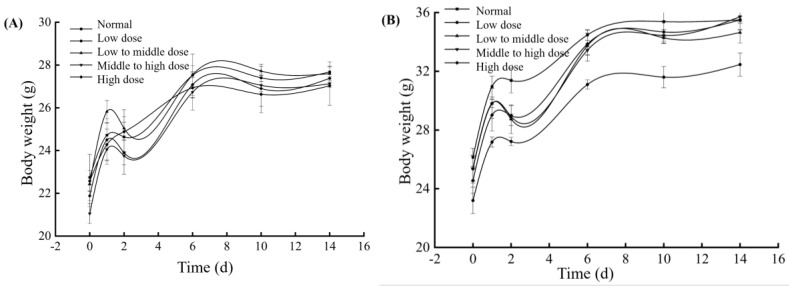
Changes in mice body weight during acute toxicity test. (**A**): female, (**B**): male.

As seen in Figure 9, the body weight of the mice in both the male and female groups increased normally during the experiment. On the 1st day after administration, the body weight of the mice in each group slightly increased, which might be caused by the retaliatory diet eating of mice after fasting. The trend of weight changes then gradually leveled off, consistent with the normal control group. The body weight of the mice showed no significant differences between the experimental groups and the normal control group (*p* > 0.05).

The organ indexes of the mice in each group are shown in Table 4. Among them, the renal index of the male mice in the low-dose group was significantly increased compared with the normal control group (*p* < 0.05). However, whether the BMSOSE can prove damaging to the kidneys of mice still needs further investigation.

**Figure 10:**
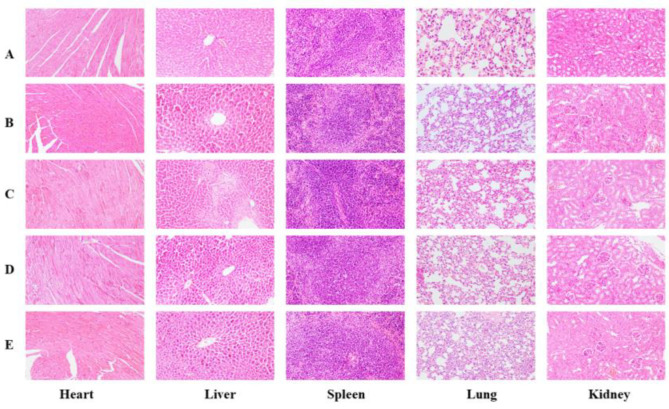
Pathological changes in male mouse organs (×200). (**A**): Normal; (**B**): Low dose; (**C**): Low to middle dose; (**D**): Middle to high dose; (**E**): High dose.

**Figure 11:**
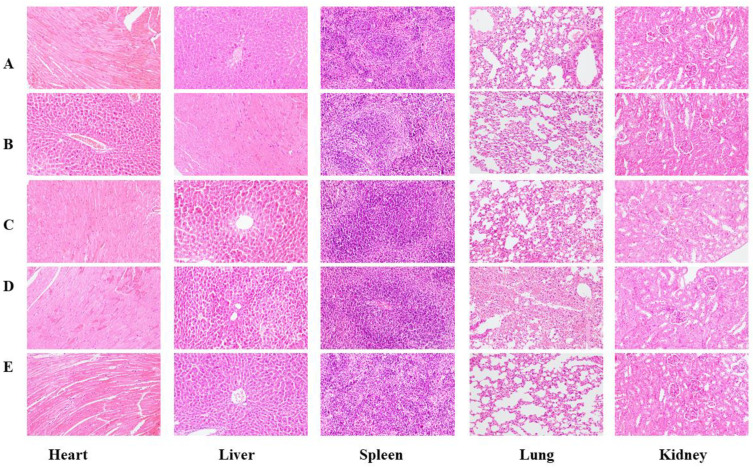
Pathological changes in female mouse organs (×200). (**A**): Normal; (**B**): Low dose; (**C**): Low to middle dose; (**D**): Middle to high dose; (**E**): High dose.

The pathological changes in the mouse organs can be observed in Figure 10 and Figure 11. Upon dissection, the organs of all mice had a normal color and were soft and fleshy, and suspicious lesions could not be directly observed. The results showed no significant difference between the control group and the experimental mice, except for sporadic and slight inflammatory infiltration in the liver and kidney of some animals in the individual dose groups, which resulted from spontaneous lesions. Therefore, the BMSOSE had no adverse effects on the development and growth of the organs in both the male and female mice.

The liver is the most important multi-functional organ in the digestive system, participating in the body’s secretion, excretion, transformation, and other functions. GPT and GOT, two critical metabolic enzymes for liver function, catalyze the amino transfer between amino acids and keto-acids. Their levels are important indexes to assess liver function damage. CR, a nitrogen-containing compound condensed by the removal of a molecule of water from creatine, is eliminated from the body by the kidney. Therefore, CR can be used as an important indicator to evaluate the glomerular filtration rate and renal function. Thus, the levels of GPT, GOT, and CR in the mice were detected. As shown in Table 5, compared with the normal control group, the indicators of the mice in each dose group injected with the BMSOSE fluctuated slightly, but all were within the normal range. Statistical analysis revealed no significant differences, suggesting that the BMSOSE did not cause damage to the liver and kidney of the mice. These results imply the safety of the BMSOSE at the tested doses.

## 4. Conclusions

The present study successfully explored the feasibility of processing a submicron emulsion (BMSOSE) using high-pressure homogenization, containing BMSO as the oil phase and egg yolk lecithin, pluronic F68, and glycerin as the emulsifier. The optimal BMSOSE contained 8% of BMSO, 1.2% of egg yolk lecithin, and 0.2% of F-68 homogenized at 600 bar and for nine cycles since it was characterized by a small spherical droplet (221.3 nm) associated with low viscosity and good fluidity. In addition, there was no significant change in the fatty acid composition of BMSO and the BMSOSE. The obtained BMSOSE showed no signs of hemolysis and toxic effects (up to an LD50 > 64 mL/kg). Overall, this SE could help incorporate and protect BSMO in the pharmaceutical, food, or cosmetics industries.

The hemolysis test showed that there was no hemolysis in the BMSOSE. The acute toxicity examination showed that the mice grew well after intraperitoneal injection of different doses of the BMSOSE. The hair of the mice was white and glossy, the appearance of the mice was no different from that of the control group, and there was no mouse casualty. After feeding for 14 days, the mice were dissected. The organs of all mice were normal in color and soft. No suspicious lesions were observed by eye observation. Compared with the normal control group, for each dose group of the mice, body weight, viscera index (heart than body, liver volume ratio, spleen volume ratio, kidney volume ratio), and serum related indicators (GPT, GOT, CR) showed no significant difference with the control group, except that the liver and kidney of individual animals in the individual dose groups appeared sporadic with slight pathological changes. Therefore, we have concluded that, under the condition of the current dose, the BMSOSE did not cause mice toxicity, and the actual non-toxic level has reached with an LD50 > 64 mL/kg. In conclusion, the BMSOSE prepared in this experiment has good stability, safety, and non-toxicity. This conclusion has a certain degree of mutual confirmation with the research results of Chung et al. [29], who found that the bitter gourd seed extract of scCO_2_ is >2000 mg/kg, which can be regarded as an almost non-toxic substance. It is crucial to note that this study, based on the practical application of bitter melon seeds, investigates the safety of the BMSOSE and offers scientific evidence for its safety evaluation in novel food assessment.

This study explores the formulation, preparation process, and safety of the BMSOSE from a practical application perspective. The development of the BMSOSE has significant potential impact in the food industry, as the BMSO is rich in CLnA, VE, polyphenols, and other beneficial ingredients for health and can be used in the development of functional foods tailored to specific health needs, such as products that regulate blood sugar and improve metabolic syndrome. The BMSOSE can also serve as a potential functional food or as an injectable emulsion for nutritional supplements. From the perspective of nutritional supplements, a BMSO injection emulsion can bypass the degradation of the digestive system and directly enter the blood circulation, which may become an efficient nutritional supplement. As a medical food, a BMSO injection emulsion may provide nutritional support for patients who cannot obtain sufficient nutrients orally. In the field of sports nutrition, the fatty acid components in BMSO may provide athletes with rapid energy supplementation and recovery, and due to the anti-inflammatory effect of BMSO, it may help alleviate muscle inflammation and injury in athletes after exercise.

However, the BMSOSE physiological activity and mechanism require further detailed research. Additionally, there is still a long way to go before the practical application of a BMSO injection emulsion. Regulatory policies for injectable emulsions vary across different countries and regions, necessitating compliance considerations based on the application location. Currently, some consumers prefer oral emulsions over injection methods. The preparation of injectable emulsions demands sterility and safety, posing challenges to production. Nevertheless, the application of injectable emulsions in the fields of food and nutritional supplements is gradually expanding, particularly in medical and sports nutrition supplements, where they exhibit significant potential. In the future, with technological advancements and changes in the consumer market, injectable emulsions are expected to be widely used in more fields. This study provided a novel idea for the development and utilization of BMSO and the utilization of natural CLnA resources, which has important practical significance in the application of seed oil.

## Figures and Tables

**Table 1:** Hemolysis test arrangement.

Tube Number	1	2	3	4	5
2% red cell suspension (RCS)	2.5	2.5	2.5	2.5	0
0.9% sodium chloride solution/mL	2.2	2.2	2.5	0	4.7
Water/mL	0	0	0	2.5	0
Bitter melon oil submicron emulsion/mL	0.3	0.3	0	0	0.3

**Table 2:** Judgment criteria of hemolysis test.

Determination Criteria for Hemolysis Result	Phenomenon
Hemolysis	The solution was clear red, and there was no cell residue at the bottom of the tube.
Part of the hemolysis	The solution was clear red or brown with a small number of cells at the bottom of the tube.
No hemolysis	The red blood cells were sinking, and the upper fluid was clear and colorless.
Erythrocyte aggregation	The red blood cells aggregated and did not disperse after vibration.

**Table 3:** Fatty acid composition of BMSO and the BMSOSE.

Fatty Acid Composition	Relative Amount (%)	
	BMSO	BMSO from BMSOSE
C16:0 (Palmitic acid)	1.68 ± 0.02	1.92 ± 0.04
C18:0 (Stearic acid)	16.8 ± 0.36	17.37 ± 0.57
C18:1 (Oleic acid)	4.34 ± 0.04	4.26 ± 0.03
C18:2 (Linoleic acid)	0.44 ± 0.01	0.42 ± 0.03
C20:0 (Arachidic acid)	6.13 ± 0.01	6.12 ± 0.07
C18:3 (α-eleostearic acid)	54.9 ± 0.60	51.83 ± 0.04
C18:3 (CLN-B)	3.63 ± 0.04	4.83 ± 0.13
C18:3 (CLN-C)	12.08 ± 0.94	13.76 ± 0.44

**Table 4:** Effect of the BMSOSE on organ indexes of mice (g, $\bar{x}$ ± s).

Gender	Group	Cardiac Index/%	Renal Index/%	Liver Index/%	Spleen Index/%
Female	Normal	0.53 ± 0.07	1.33 ± 0.06	4.18 ± 0.06	0.38 ± 0.04
	Low dose	0.60 ± 0.06	1.25 ± 0.06	4.18 ± 0.36	0.41 ± 0.11
	Low to middle dose	0.53 ± 0.12	1.33 ± 0.04	4.75 ± 0.66	0.40 ± 0.04
	Middle to high dose	0.58 ± 0.00	01.22 ± 0.15	4.78 ± 0.26	0.50 ± 0.02
	High dose	0.62 ± 0.04	1.35 ± 0.09	4.34 ± 0.29	0.38 ± 0.04
Male	Normal	0.51 ± 0.05	0.80 ± 0.23 ^b^	4.56 ± 0.19	0.38 ± 0.08
	Low dose	0.52 ± 0.06	1.60 ± 0.16 ^a^	4.90 ± 0.29	0.45 ± 0.06
	Low to middle dose	0.54 ± 0.11	0.84 ± 0.12 ^b^	4.90 ± 0.47	0.44 ± 0.07
	Middle to high dose	0.54 ± 0.11	0.90 ± 0.08 ^b^	4.61 ± 0.17	0.38 ± 0.05
	High dose	0.54 ± 0.07	0.83 ± 0.08 ^b^	4.63 ± 0.41	0.36 ± 0.009

Different lower-case letters indicate significant differences between the number of passes (*p* < 0.05).

**Table 5:** Effect of the BMSOSE on serum indexes of mice (g, $\bar{x}$ ± s).

Gender	Group	GPT/(U/L)	GOT/(U/L)	CR/(μmol/L)
Female	Normal	16.51 ± 4.07	22.04 ± 7.00	72.99 ± 8.18
	Low dose	17.76 ± 2.17	25.04 ± 4.60	80.18 ± 5.20
	Low to middle dose	14.69 ± 3.04	28.67 ± 1.20	80.08 ± 12.47
	Middle to high dose	16.95 ± 3.50	22.49 ± 8.39	88.75 ± 9.10
	High dose	13.35 ± 3.55	17.57 ± 3.04	77.26 ± 7.65
Male	Normal	21.36 ± 5.89	21.03 ± 4.06	74.20 ± 1.84
	Low dose	20.86 ± 7.08	24.99 ± 5.09	75.75 ± 4.13
	Low to middle dose	16.08 ± 1.67	31.27 ± 7.74	90.92 ± 2.29
	Middle to high dose	17.82 ± 8.11	24.59 ± 3.49	80.94 ± 4.99
	High dose	20.41 ± 1.99	20.86 ± 5.13	74.71 ± 7.08

## Data Availability

The data that support the findings of this study are not publicly available due to privacy or ethical restrictions.
